# When the statistical MMN meets the physical MMN

**DOI:** 10.1038/s41598-019-42066-4

**Published:** 2019-04-03

**Authors:** Vera Tsogli, Sebastian Jentschke, Tatsuya Daikoku, Stefan Koelsch

**Affiliations:** 1 University of Bergen, Department for Biological and Medical Psychology, Postboks 7807, 5020 Bergen, Norway; 2 University of Bergen, Department of Psychosocial Science, Postboks 7807, 5020 Bergen, Norway; 3 Max Planck Institute for Human Cognitive and Brain Sciences, Stephanstr. 1a, 04103 Leipzig, Germany

**Keywords:** Cognitive neuroscience, Learning and memory, Psychology

## Abstract

How do listeners respond to prediction errors within patterned sequence of sounds? To answer this question we carried out a statistical learning study using electroencephalography (EEG). In a continuous auditory stream of sound triplets the deviations were either (a) statistical, in terms of transitional probability, (b) physical, due to a change in sound location (left or right speaker) or (c) a double deviants, i.e. a combination of the two. Statistical and physical deviants elicited a statistical mismatch negativity and a physical MMN respectively. Most importantly, we found that effects of statistical and physical deviants interacted (the statistical MMN was smaller when co-occurring with a physical deviant). Results show, for the first time, that processing of prediction errors due to statistical learning is affected by prediction errors due to physical deviance. Our findings thus show that the statistical MMN interacts with the physical MMN, implying that prediction error processing due to physical sound attributes suppresses processing of learned statistical properties of sounds.

## Introduction

Statistical learning lies at the heart of our everyday lives. It is a mechanism that enables individuals to detect statistical patterns in the environment without explicit intent to learn. Statistical learning comes under the umbrella term of implicit learning^1^. It has been investigated using a range of stimulus types (for reviews, see refs ^2,3^) such a syllables^4^, tones^5^, sung language^6^, and visual stimuli^7^, reflecting the domain-general aspect of this mechanism^8^. However, most statistical learning studies have focussed on the domain of language acquisition and specifically on word segmentation, which is based on the principle that adjacent syllables within a word have greater transitional probabilities compared with adjacent syllables between words. A number of studies explored the neural correlates of statistical learning assessing event-related potential (ERP) or event-related magnetic field (ERF) components such as the N400^9–13^ or the N100/N100m^9,13–15^. Earlier components have also been reported such as auditory brainstem responses (ABR)^16^ or the P50m^17,18^.

Only recently, some studies using statistical learning paradigms began to explore neural correlates beyond those related to word segmentation (for a review, see ref.^3^), using the “Mismatch Negativity (MMN)” as a neurophysiological marker of processing “statistical deviance” (by comparing low and high transition probabilities). The main question in those studies was how the human brain responds to surprising events that violate learned regularities, and especially when these irregularities occurred within a word or a sound pattern (ex: “beautiful cactus” vs “beautiful cacti” or “pretty baby” vs “pretty babies”). Koelsch *et al*.^19^, generated such irregularities by manipulating the transitional probability between the second and the third tone within tone triplets. They found that more unexpected events (i.e., with low transitional probability) elicited a larger mismatch response. The authors referred to this ERP effect as statistical MMN (sMMN). Another study by Moldwin *et al*.^20^, observed that the MMN elicited by equiprobable deviant tones was stronger when the violation occurred within rather than between melodic patterns. Thus, the MMN was larger for violations occurring in positions with high transition probability, within a melodic pattern, compared with positions with low transition probability such as between melodic patterns. The reported MMN could also be regarded as sMMN since it reflects learning of probabilistic properties of sounds. François *et al*.^21^, found that violations to word structure (regular: ABC vs irregular: CBA) disrupted the transitional probabilities of the syllables within the word, evoking negativities within the time windows of the MMN/N200 that had a dominance over fronto-central scalp regions. It appears that very few statistical learning studies with EEG have investigated effects beyond perception of word boundaries. Therefore, the primary scope of our study was to investigate neural correlates of statistical learning by varying transitional probabilities not only between the words but also within the word.

Another question that remains unexplored in statistical learning studies is if and how the statistical manipulation of physical attributes of the audio stimuli, such as loudness, spatial location or frequency, interacts with learning. Several studies have investigated how the brain responds to changes on the physical or abstract features of the sound, reporting the physical MMN (phMMN) or the abstract feature MMN^22–26^. However, to our knowledge, there is no study so far exploring the statistical manipulation of transitional probabilities and physical attributes of sounds within the same paradigm. Thus, a secondary scope of our study was to provide insights into a possible interaction between the sMMN (elicited by transition probabilities) and the phMMN (elicited by a sound location change). If an interaction can be observed between statistical and physical MMN, this would imply that processing of the transitional probabilities and of the location change of the sound overlap. In other words, the mechanism engaged for change detection would be the same for both types of MMN. In a study by Koelsch *et al*.^27^, it was found that the amplitude of LAN, a component elicited by morphosyntactic violations in speech, was not affected by the presence of physical violations. This indicates that the underlying cognitive processes are independent. However, compared to the LAN with its higher latency (about 100 to 300 ms), the statistical and the physical MMN (explored in our study) occur within a similar time window. This, in turn suggests that an interaction between these two components is more probable.

The current study will allow us to examine processing related to “sensory” features, such a sound location, and more “structural” features of the stimuli, such as transitional probability. Assuming that MMN neural traces reflect how stimuli are represented in auditory memory^28^, then the different stimuli features should be encoded differently. Of greater interest is the co-occurrence of sound location change and transitional probability deviation and the way these will be encoded in a unitary representation as reflected in the MMN. It is important to note that the critical difference between the phMMN^22^ and the sMMN is that the former relies on operations of the auditory sensory memory and sensory-memory-representations that are updated instantly^29,30^. The latter, in contrast, relies on memory representations that are formed due to a learning process that leads to memory representations going beyond the capabilities of the sensory memory. To illustrate this, a deviance in terms of sound location would elicit instantly a phMMN caused by a mismatch of the recent auditory input compared with the deviant sound, as processed in the auditory sensory memory. In contrast, deviations in terms of learned transitional probabilities elicit a sMMN under the condition that (implicit) knowledge of the statistical regularities was acquired. We expected the physical deviance (elicited by a change in sound location) compared with the statistical deviance (elicited by low in comparison to high transitional probability) would be more prominent and easier to process (i.e., be cognitively less demanding). Additionally, from the perspective of predictive coding^31^ both deviant events (i.e., statistical and physical) would generate prediction errors that the brain would try to minimise. Most probably, the physical prediction errors would be easier to resolve due to the higher saliency of the exteroceptive stimuli. As a consequence, we expected that the phMMN would have a larger amplitude compared to the sMMN and that the phMMN would attenuate the sMMN.

A third aspect of our study was to develop an experimental paradigm suitable for investigations with children. So far, most of the studies employing statistical learning paradigms focused on the segmentation of the auditory stream into words or word-like units. The age of the participants in those studies ranged from infants to adults. For example, several statistical learning studies with children have investigated effects of musical training^10,11^, or reading ability^32^ in children with typical development, or differences between children with typical development and atypical language development^33,34^. It appears that children with language disorders have impaired performance in statistical learning tasks^35^. However, given that these studies focused on word segmentation processes, it remains to be explored whether and how the sMMN, reflecting the cognitive processing of different transition probabilities within a word, is able to differentiate between children with typical and with impaired language development.

**Figure 1:**
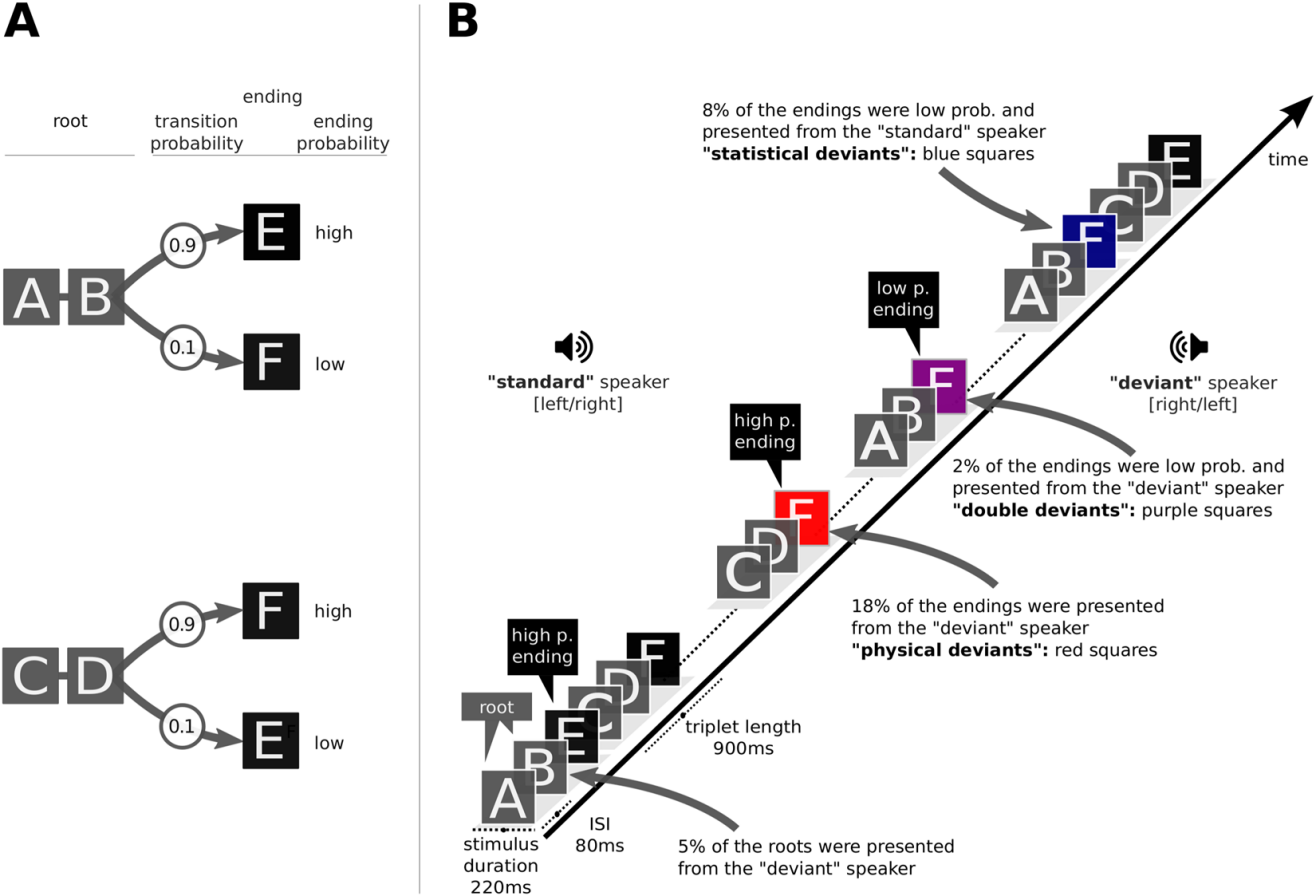
Experimental Paradigm. (**A**) The four triplets generated from 6 pairs of sounds. The first two items of the triplet are named root and the last item is name ending. Statistical deviants were created by varying the transition probability from root to ending within two levels, high (p = 0.9) and low (p = 0.1). Triplet roots (AB or CD) are occurring with a constant transitional probability (p = 0.5) from any of the triplet endings (E or F). (**B**) The auditory stream of pseudorandomly concatenated triplets with standard ending triplets (letter in black box), statistical deviant ending triplets (letter in blue box), physical deviants (letter in red box) and double deviants (letters in purple box). Physical deviants were generated by switching speaker.

To this end, the experimental paradigm of the current study was developed from a previous study by Koelsch *et al*.^19^. In this experiment, a continuous stream of sounds, organised in triplets, was presented (see Fig. 1A). The first two sounds of the triplet, denoted as root, can be considered as one unit (the transition probability between the first and the second sound was always 1). The transition probability (TP) between the root and the last sound of the triplet was manipulated to be either low (TP = 0.1), intermediate (TP = 0.3), or high (TP = 0.6). The current experiment follows the same rationale but simplified the paradigm: The transition probability of the last sound of the triplet had only two steps, either high (TP = 0.9) or low (TP = 0.1). Given that the two sounds within the root always appeared together, our paradigm represents a 1st-order Markov model or bigram model with strictly 2-local distribution^36^. Also, the transition probability between the words was constant (TP = 0.5). The current paradigm furthermore introduces a physical deviance, elicited by location change of the sound. Standard sounds would be presented from a default direction (either right or left speaker) whereas deviant sounds would be presented from the opposite direction (either left or right speaker).

We hypothesised that low probability events (i.e., triplet endings with low vs. high transitional probability) would elicit a sMMN. The second hypothesis was that physical deviants would elicit a phMMN. Third, we tested for an interaction of transition probability and physical deviance (i.e., an interaction between statistical and physical MMN), and hypothesised that the physical deviants would diminish the effect of the statistical deviants.

## Results

### Behavioral data

#### Familiarity Test

Participants achieved an overall score of 52.5% correct responses (SEM = 2.5%) and their performance did not differ significantly from chance level (*p* = 0.38). Only one participant (out of 21) reported during debriefing that she became aware of the statistical regularities underlying the arrangement of the stimuli; she also performed above chance level (64% correct responses).

#### Cover Task

Participants detected 99.5% of the (higher-pitched) target sounds successfully.

### Electroencephalographic data

The results for the ERPs will be presented first for the triplet endings and subsequently for the triplet roots.

#### ERPs of Triplet Endings

**Figure 2:**
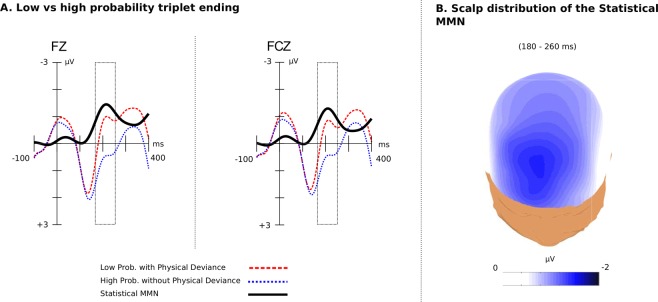
The statistical MMN (sMMN) over the three blocks. (**A**) The sMMN (black line) generated due to the violation of the standard transitional probability of the triplet ending from high TP = 0.9 to low TP = 0.1 and as captured at electrodes Fz and FCz. Last tone (triplet ending) onset is at 0 ms and has a duration of 300 ms. ERPs are baseline corrected 100 ms prior to the onset of the trigger. (**B**) The scalp distribution of the sMMN over the time window (180 to 260 ms) was maximal over the frontal-midline region.

Statistical Deviants. Both low and high probability endings elicited a P1-like positivity maximal at around 140 ms, which did not differ between high and low probability endings (*p* = 0.16), followed by a negative deflection at around 200 ms that was larger for low probability endings compared with the high probability endings (see Fig. 2A). The amplitude difference (between high and low probability endings) is denoted as statistical MMN (sMMN). It had a size of −1.50 *μ*V at the peak maximum (latency: 210 ms). This effect was analysed statistically with an ANOVA for the time window of 180 to 260 ms relative to the onset of the triplet ending with factors transition probability (high vs. low), scalp area (anterior, central and posterior), lateralisation (left, midline and right) and block (1 to 3). It revealed a significant effect of transition probability (${F}_{(1,20)}=35.54$, $p < 0.0001$, Cohen’s *d* = 5.10), no significant effect of block (*p* = 0.30), no interaction between probability and block (*p* = 0.59), thereby confirming our hypothesis that low probability endings would elicit a greater negativity. Furthermore, the following significant interactions were found: (a) transition probability and scalp area (reflecting that the sMMN effect was largest at anterior and diminishes towards posterior sites; (${F}_{(2,19)}=6.9$, $p < 0.006$), (b) transition probability and lateralisation (reflecting that the sMMN was largest at midline sites; (${F}_{(2,19)}=23.13$, $p < 0.0001$) and (c) block, scalp area and lateralisation (${F}_{(4,17)}=4.19$, $p=0.015$). A linear trend was found for the interaction between transition probability and scalp area (*p* = 0.001). This reflects that the amplitude difference between low and high transition probability diminished from anterior towards posterior scalp sites. Pairwise comparisons for effect of triplet ending across all ROIs showed that the effect was significant (*p* < 0.05) in all of them (see Table 1). However, the effect was maximum in the frontal-midline region (with the maximum observed at electrode Fz, see Fig. 2B).

**Table 1:** Mean amplitudes of the statistical MMN (comparing the difference in the neurophysiological brain response to low minus high transition probability endings; in *μ*V) averaged over the participants.

	Left hemisphere		Middle hemisphere		Right hemisphere		ROI means	
	Mean difference (low-high)	*p*	Mean difference (low-high)	*p*	Mean difference (low-high)	*p*	Mean difference (low-high)	*p*
Anterior	−0.87	<0.001	−1.28	<0.001	−0.97	<0.001	−1.04	<0.001
Central	−0.59	<0.001	−1.01	<0.001	−0.58	<0.05	−0.73	<0.001
Posterior	−0.46	<0.05	−0.65	<0.05	−0.30	<0.05	−0.47	<0.05

Pairwise comparisons examined how the effect of transition probability evolved across the experiment (see Fig. 2C). The analysis showed that low probability ending elicited a significant sMMN already in the first block (*p* = 0.002), increased in size for the second (*p* = 0.001), and then decreased for third block of the experiment (*p* = 0.028).

#### Physical Deviants

**Figure 3:**
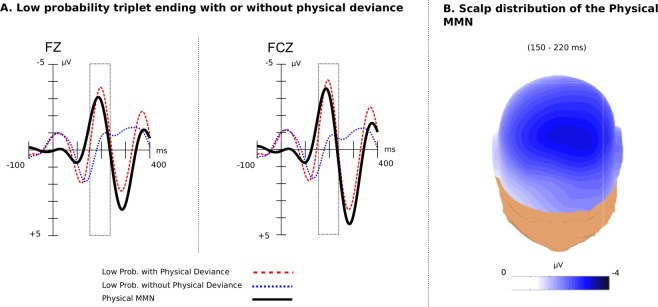
The physical MMN (phMMN). (**A**) The phMMN (black line) generated due to the violation of sound location of triplet ending from the “standard” to the “deviant” side, as captured at electrodes Fz and FCz. Last tone (triplet ending) onset is at 0 ms and has a duration of 300 ms. ERPs are baseline corrected 100 ms prior to the onset of the trigger. (**B**) The scalp distribution of the phMMN over the time window (150 to 220 ms) was maximal over the frontal and central midline regions.

To investigate the effect of the physical deviance, we compared the ERP responses to triplet endings either containing physical deviance or not. The analysis compared the brain response to triplet endings where low transition probability co-occurred with the sound being presented either from the standard or the opposite direction. For the effect of physical deviance on the high-probability triplet ending see the Supplementary Fig. 5. As shown in Fig. 3A, triplet endings with physical deviance elicited a positive-going wave with a latency of about 100 ms (P1) and a phMMN reaching the maximum amplitude of −3.65 *μ*V at around 180 ms over central-midline electrodes. The phMMN was followed by a distinct P3b (see Supplementary Fig. 6). The effect of physical deviance appeared to be larger and earlier (amplitude: −3.65 *μ*V, latency: 180 ms) compared with the effect of statistical deviance (amplitude: −1.50 *μ*V, latency: 210 ms).

An ANOVA with the factors physical deviance (with vs. without location change), scalp area (anterior, central and posterior), lateralisation (left, midline and right) and block (1 to 3) over the time window of 150 to 220 ms after the onset of the triplet ending showed significant effect of physical deviance (${F}_{(1,20)}=38.90$, $p < 0.0001$, Cohen’s *d* = 7.26). No significant effect of block was found or any interaction between block and triplet ending.

Triplet endings with location changes elicited consistently larger negativities throughout the course of the experiment even though the amplitude size diminished over blocks (see Fig. 3C). However, the two ending types differed significantly within the first (*p* = 0.001), the second (*p* < 0.0001) and the third block (*p* = 0.014) of the experiment.

With respect to the scalp distribution, the physical deviance led to an amplitude difference (*p* < 0.05) across all ROIs except from the posterior right one. Furthermore, it reached maxima in the frontal and central midline electrodes (and a maximum amplitude difference at FCz, see Fig. 3A).

The P1 time window (80 to 180 ms) was assessed using an ANOVA with the factors physical deviance (with vs. without location change), scalp area (anterior, central and posterior), lateralisation (left, midline and right) and block (1 to 3). It revealed a significant effect of physical deviance (${F}_{(1,20)}=38.90$, $p < 0.00016$, Cohen’s *d* = 3.42), no significant effect of block or any interaction involving block and physical deviance. Yet, the physical deviance affected the ERP responses in a decreasing rate; it was significant only during the first block (*p* = 0.028). The distribution of the effect was not symmetrical but rather left-lateralised and diminished towards right scalp sites (${F}_{(2,40)}=3.54$, $p=0.038$).

#### Interaction of probability and change location

To investigate the interaction between the two types of deviances, (i.e.^1^, the varying transition probability of the triplet ending and^2^ the presence or absence of location change), we conducted an ANOVA with transition probability (high vs. low), physical deviance (with vs. without location change), scalp area (anterior, central and posterior), lateralisation (left, midline and right) and block (1 to 3) in a the time window from 160 to 260 ms after the onset of the triplet ending.

**Figure 4:**
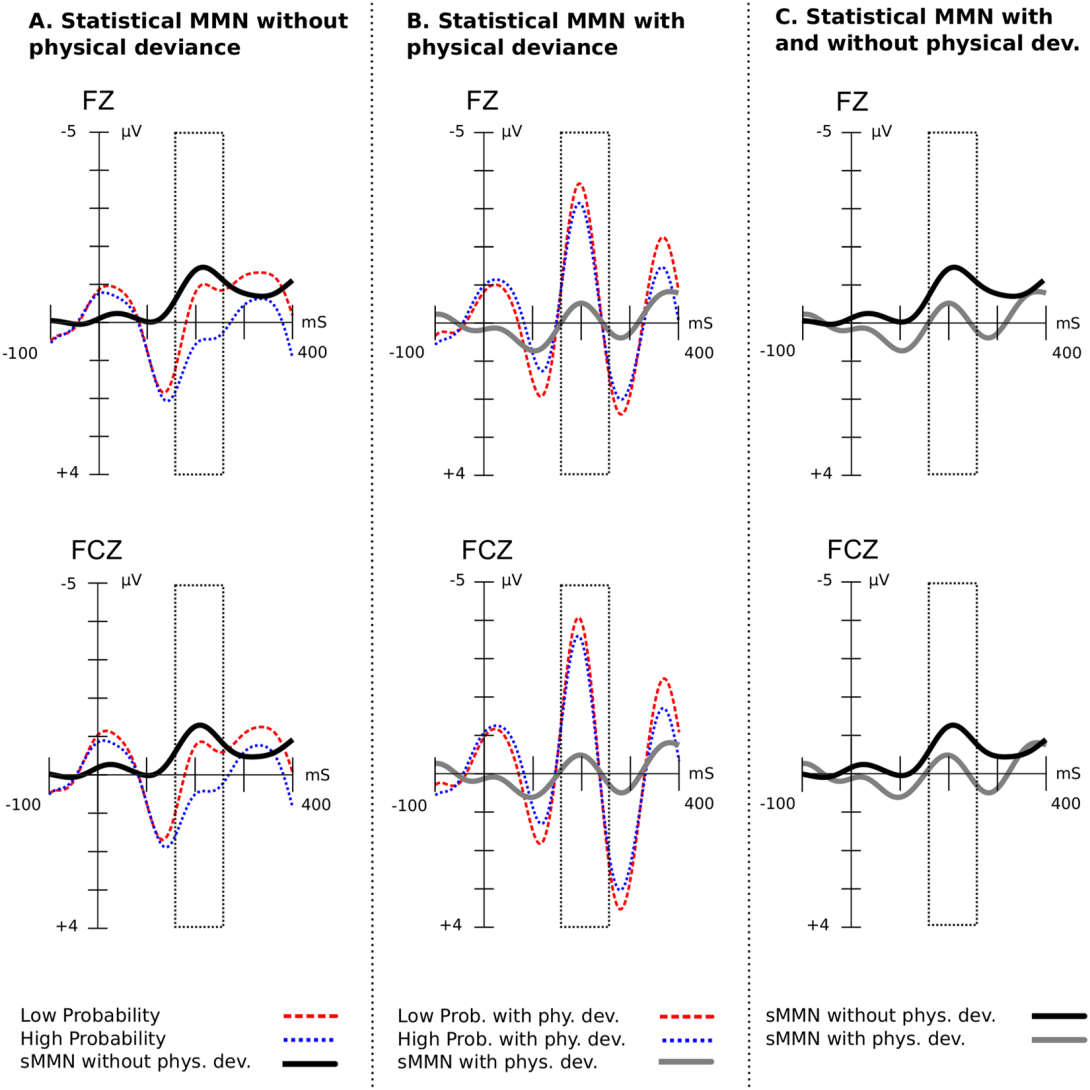
Interaction of transition probability and physical deviance. Triplet ending onset is at 0 ms and has a duration of 300 ms. ERPs are baseline corrected 100 ms prior to the onset of the trigger. (**A**) The statistical MMN (black line) without physical deviance, as captured at electrode Fz and FCz. The trace depicted for electrodes Fz and FCz is identical to the one in Fig. 3A. (**B**) The statistical MMN (gray line) with physical deviance. (**C**) Separate traces for the statistical MMN with or without physical deviance. At the presence of physical MMN the statistical MMN diminishes.

The analysis revealed a significant effect of probability (${F}_{(1,20)}=14.14$, $p=0.001$, Cohen’s *d* = 3.14), of physical deviance (${F}_{(1,20)}=35.02$, $p < 0.0001$, Cohen’s $d=6.40$), and interaction of these two (${F}_{(1,20)}=4.95$, $p=0.038$). Pairwise comparisons showed that at the presence of physical deviance the effect of statistical deviance almost disappears ($p=0.205$; see Fig. 4B). Statistical deviance produced a significant effect ($p < 0.0001$) only under the condition that there was no physical deviance.

#### ERPs of Triplet Roots

To examine whether the continuous auditory stream was segmented into discrete triplets, we examined the ERP responses to the triplet roots (i.e., the first two triplet items). We hypothesized that any difference in the ERP responses within a late N1 window (170 to 250 ms) between the first two triplet items should reflect such segmentation effect. This analysis assessed the first and the second sound (both coming from within triplets that were preceded by standard triplets; i.e., those having high probability endings and no physical deviance).

An ANOVA with factors position (first and second sound), scalp area (anterior, central and posterior), lateralisation (left, midline and right) and block (1 to 3) for the N1 time window (from 170 to 250 ms) revealed no significant effect of position (*p* = 0.496), no significant effect of block (*p* = 0.225) but a significant interaction between these two (${F}_{(2,40)}=3.79$, $p=0.031$). Therefore, typical word segmentation effect on the first item could not be observed in the current study.

**Figure 5:**
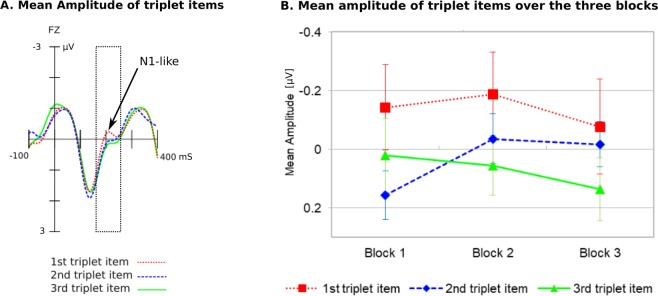
N1-like wave for the three items of the triplet. (**A**) Triplet item onset is at 0 ms with a duration of 300 ms. ERPs are filtered 100 ms prior to the onset of the trigger. There is no typical segmentation effect over the window 170 to 250 ms. (**B**) The mean amplitudes of the triplet items over the blocks. There is no significant difference between them.

Figure 5 depicts ERP responses, separately for each of the triplet tones. Each item elicited a P1-like positivity at around 140 ms, and N1-like negativity at about 200 ms. This N1-like negativity differed between the three triplet items.

The ANOVA for the P1 time window from (100 to 200 ms after the onset of the triplet item) revealed no significant effect of position (*p* = 0.390), a no significant effect of block (*p* = 0.670) neither a significant interaction between these two (*p* = 0.160).

## Discussion

Low-probability triplet endings elicited a sMMN, i.e. a larger fronto-central negativity within a time window between 180 to 260 ms (compared with high probability triplet endings). The observation of this sMMN is in accordance with previous studies that manipulated the transitional probability of deviants and reported similar ERP effects^15,17,19^. Thus, the present study confirms that the sMMN can be used to investigate neurophysiological effects due to the acquisition of knowledge about transition probabilities based upon statistical (implicit) learning. Importantly, the sMMN (elicited by the low-probability triplet endings) was due to the transitional probability with which the triplet endings occurred, and not due to any acoustical or perceptual differences. Thus, we can exclude that the sMMN received any contributions from a physical or abstract feature MMN. Moreover, due to the fact that knowledge about the underlying statistical regularities is not acquired instantly (as is the case with physical features of the sound), this statistical mismatch involves memory representations beyond the capabilities of sensory memory.

Although the ERP results indicate that participants acquired implicit knowledge about the regularities underlying the arrangement of tones within the triplets, they did not exhibit explicit knowledge: The performance in the behavioural part (assessing familiarity with the more frequently played sequence containing the high transition probability continuation) was only marginally above chance level and did not reach significance. Participants were also not able to express verbally what they learned when explicitly asked to do so. Therefore, it appears that the statistical learning during the experiment, as indicated by the sMMN, was implicit. The discrepancy between behavioral and neurophysiological evidence is not unusual in implicit learning studies^17,19,37^. However, perhaps an easier behavioral task could have revealed learning effects (as evidenced in the neurophysiological data). Future studies might consider using also “non-words”, i.e., triplets that did not occur during the exposition phase (such as FEA, FEB, EFC and EFD), in addition to triplets that were presented during the exposition phase (ABE, ABF, CDF and CDE).

When triplet endings were presented in a different spatial location (with low probability of the physical location change), these location deviants elicited effects within the P1-time window, followed by a clear phMMN (which appeared to have a shorter peak latency than the sMMN)^38^. These observations suggest that sound location change identification is an earlier process compared to statistical deviance detection (at least during the early phases of learning, i.e. within the first hour of learning, as investigated in the present study). In contrast to the statistical deviants, the location changes were rather salient (with a rather large angle of 60° location change)^39^. This difference in saliency is also reflected in the observation that the amplitude of the phMMN was larger than the amplitude of the sMMN. The scalp distribution of the present MMN was maximal over midline electrodes within fronto-central regions, which is in agreement with previous studies^25,40^.

Interestingly, the co-occurrence of statistical and physical deviance did not produce an additive effect, but an interaction. As shown in Fig. 4, the sMMN elicited by deviants with low transition probability diminished in the presence of physical deviance. In other words, the effect of statistical deviance is smaller when co-occurring simultaneously with a location change. This interaction suggests that neural substrates underlying the processing of transition probability and physical deviance overlap at least partly.

To explain this interaction we refer to the four necessary processes for MMN elicitation as presented in Schröger^41^ and Koelsch^42^. Assuming that the elicitation of an MMN requires a pre-established representation of the stimuli in the auditory sensory memory (ASM)^28^, the formation of these representations is regarded as the first phase. In principle, at this early phase, mainly sensory processes such as feature extraction or detection of source location are carried out. The outcome is a unitary stimulus representation of the auditory event where different features of the stimuli are integrated^28^. Therefore, in our study, we may assume that three different types of representations were formed for sMMN, phMMN and double deviance MMN. In the second phase, a model is established that embeds all acoustical or structural regularities of the input. In our experiment participants established a model with certain physical features that are perceived in real time (i.e., standard vs. deviant sound location). In contrast, transition probabilities between triplet items require some time to be learned. This knowledge is reflected by the physical and statistical MMN when tested solely. In a third phase, upon establishment of a model, predictions are made about specific future auditory events. In the fourth phase, the model is tested; if the newly incoming stimuli mismatch with the model predictions, an MMN is triggered. Based on our findings we propose that the elicitation of the MMN (whether it is due to statistical, physical or double deviance), relies on partly the same mechanism, but is elicited by different input representations. The interaction suggests an overlap of neural resources during prediction formation (third phase) and comparison (fourth phase). By contrast, stimuli representation formation (first phase) relies on different neural populations. We propose that statistical deviance encoding engages more top-down operations, compared with physical or double deviance encoding, since it requires some time for learning the underlying regularities. Particularly in the case of double deviance, the prominent physical deviance dominates the formation of the stimulus representation along with the predictions, and accordingly affects the comparison process more significantly. Along the same line, our results also support the notion that neural traces in MMN generation reflect the perceived stimuli rather than the actual properties of the stimuli^28^.

An important question that remains to be answered is whether attention affects the interaction of statistical and physical deviance. To our knowledge, there is no study investigating the role of attention for the elicitation of the sMMN or the interaction of statistical and physical MMN. Therefore, we cannot exclude the contingency that processing of physical deviances interferes, via attention or not, with processing of learning statistical regularities. However, the finding that the sMMN is influenced by a physical deviance remains; but we are not able to tell if the learning is actually disturbed by the physical deviance. Hence, the interference effect of attention to the sMMN remains to be specified.

Within the predictive coding framework the deviances are viewed as prediction errors which are ascending the auditory cortical hierarchies to evince better predictions^43^. Presumably, prediction errors due to statistical deviances have to ascend higher the hierarchy to be resolved compared with prediction errors due to physical deviances. On the basis that physical deviants are more precise and predictable, they also gain greater attention. Thus, attending to the physical deviants suppresses processing of statistical cues by ignoring evidence for statistical prediction errors. Although physical prediction errors are probably resolved lower in the auditory hierarchy than the statistical prediction errors, our results indicate that there is some overlap in the levels of prediction error processing in the auditory hierarchy.

This study was primarily about responses related to processing of high vs low probability, and less to the perception of word boundaries. Thus, along with the ERP responses to deviances, we also examined whether the participants segmented the auditory stream into discrete triplets. For this purpose, we compared the N1-like wave as generated by the first two triplet items. We found that the amplitude between the first and the second item did not differ significantly. Thus, we did not obtain a typical word segmentation effect in our study. This may be due to the fact that the transitional probability between words was relatively high (TP = 0.5), thus, not creating a clear statistical boundary. Interestingly, in the study by Koelsch *et al*.^19^ where the transitional probability between words was lower (TP = 0.33) there was a significant word segmentation effect (see Supplementary Fig. 1) Thus, we may draw the conclusion that a transitional probability between words of 0.5 is not sufficiently low to produce a clear word segmentation effect. Nonetheless, as it was noted in the introduction, the primary aim of this study was not on word segmentation per se, but to give a deeper understanding on the mechanisms employed during statistical learning using a novel paradigm.

The presented paradigm seems to be appropriate to conduct studies with children, both with typical and impaired language development. First, the stimuli are of musical rather than syllabic nature. This ensures that statistical learning would not be biased by interindividual differences in phoneme perception skills. Second, the experimental paradigm features a child-appropriate duration of an experimental session. Third, in addition to word segmentation learning it incorporates processing of high- and low-probability events which is suited to investigate the so-called sMMN. Fourth, it allows comparison of responses due to statistical learning (sMMN) with responses due to sensory learning, i.e. acoustical mismatch detection of the auditory sensory memory as reflected in the “physical mismatch negativity” (phMMN, we used a location MMN to achieve this). Fifth, it meets the requirement to investigate possible interactions between statistical and sensory learning (i.e., between the sMMN and the phMMN).

## Conclusion

Our study replicates the sMMN, an ERP component triggered by events that deviate from regularities acquired within a statistical (implicit) learning paradigm. The novelty of our paradigm lies on the fact that we combined within one experimental paradigm the violation of statistical expectancies (i.e., implicit knowledge about the different transition probabilities at the last tone of a sound triplet acquired over the course of the experiment) with a violation of expectancies regarding physical features (i.e., a change in sound location). As expected, both types of deviance elicited clear ERP effects (i.e., either a sMMN or a phMMN). The current paradigm seems promising for future studies involving children with typical and non-typical language development. This might elucidate neural substrates involved in specific language impairment, and could potentially lead to the design of a diagnostic tool for language impairments^44^.

A new finding of our study was that the sMMN interacts with the phMMN: The sMMN was diminished in the presence of a location change. This finding suggests that the elicitation of statistical and physical MMN share neural resources, mostly with regard to processing resources, rather than resources for representations. Additionally, it indicates that the brain responds selectively to deviations that are easy to detect and consequently do not require learning.

## Methods

### Participants

21 adults (12 females; mean age = 22.43 years, SD = 2.39) were recruited at the University of Bergen. None of the participants had hearing impairments, history of neurological disease, or musical training more than 2 years besides regular school lessons (according to self-report). All participants were compensated (200 NOK, approx. 20 EUR) at the end of the experiment. The study was carried out in accordance with the guidelines of the Declaration of Helsinki, and approved by the Regional Committee for Medical and Health Research Ethics for Western Norway (Reference Number: 2014/313). Participants provided written informed consent before the experiment.

### Stimuli

#### Sound triplets

In order to form the triplets we created six sounds. Each sound was a combination of a Shepard tone and a percussion sound. Shepard tones^45^ were employed in order to reduce any percept of pitch along with any auditory grouping based on pitch. We generated six Shepard tones for six frequencies (F3: 174.61 Hz, G3: 196.00 Hz, A3: 220.00 Hz, B3: 246.94 Hz, C#4: 277.18 Hz and D#4: 311.13 Hz) where each tone resulted from the superposition of nine sinusoidal components spaced one octave apart. These six Shepard tones were combined with six percussive sounds (surdo, tambourine, agogo bells, hi-hat, castanet and woodbloc, which were downloaded from http://www.philharmonia.co.uk/explore/sound_samples. The Philarmonia Orchestra website). All sounds were sampled at 44100 Hz and normalised based on the RMS amplitude so that they were matched in overall loudness. Each sound had a duration of 220 ms, including a fade in of 10 ms and fade out of 20 ms. The interstimulus interval was 80 ms (thus, the inter-onset interval was 300 ms). The six sounds, corresponding to the letters A to F (see Fig. 1), were combined into triplets. Specifically, sounds A, B, C and D were combined in two (AB and CD) in order to form the “root” of the triplet. The word “root” was used to refer to the first two items of the triplet. Sounds E and F were used for the last position or item of the triplet. Thus, we obtained four unique triplets (Fig. 1).

For the practice trials before the experiment, a second set of six sounds was created. These sounds were created similar to the sounds of the main experiment, but differed in frequency of the Shepard tones (E3: 164.81 Hz, F#3: 184.99 Hz, G#3: 207.65 Hz, A#3: 233.08 Hz, C4: 261.62 Hz and D4: 293.66 Hz) and in which percussive sounds were used (woodblock, tambourine, agogo bells, castanet, hi-hat and bass drum).

Finally, an additional sound was created (C#5: 554.37 Hz, not combined with a percussive sound) to serve as target sound for the cover task that participants had during practice trials and the experiment.

Importantly, the arrangement of sounds (A to F) was permutated across participants as a way to guarantee that possible acoustical differences between sounds would not bias the brain responses of interest.

#### Stimulus Location

In order to generate physical deviants, the location of the sounds stimulus was manipulated, featuring a spatial location change of 60° angle in the azimuthal plane: If the standard stimuli were presented from the direction of one speaker, the physical deviants were presented from the other one. The stimulus location was arranged as follows: The sound of the triplet root (A to D) was presented in 95% of the times from the “standard” and 5% of the times from the “deviant” location. The last sound of the triplet was presented in 80% of the times from the “standard” side, and 20% of the times from the “deviant” side. Only sounds at the last position were evaluated when assessing physical deviance. “Standard” and “deviant” location was balanced across blocks and counterbalanced between participants whether they would have left or right as preferential (standard) direction for the first block.

#### Triplet Endings

Triplets endings differed in respect to (1) the frequency of occurrence of their ending within the experiment block and (2) the location of their ending (Table 2). Therefore, four categories of triplet ending occurred:

1. *Standards*: represented the 90% of the triplets, featured a high transition probability ending (p = 0.9) and were presented from the “standard” location.
2. *Transitional Probability Deviants*: 10%, featured a low transition probability ending (p = 0.1) and were presented from the “standard” location.
3. *Physical Deviants*: 20%, featured an ending presented from the “deviant” location.
4. *Double Deviants*: transition probability and physical deviants represented 2% of the triplets. They featured a low transition probability ending and were presented from the “deviant” location.

**Table 2:** Distribution of transition probabilities and location change in triplet endings.

	Triplet Ending	
Location	High Probability (p = 0.90)	Low Probability (p = 0.10)
Standard (p = 0.80)	Standards (p = 0.72)	Statistical Deviant (p = 0.08)
Deviant (p = 0.20)	Physical Deviant (p = 0.18)	Double Deviant (p = 0.02)

#### Triplet Streams

400 triplets were pseudorandomly concatenated into pause-free streams or blocks of about 7 min duration each. Triplets were presented in a pseudorandom order so that triplets from the low probability set were separated by at least three triplets from another set. Triplet roots (AB or CD) followed any of the two triplet endings (E or F) with a constant transitional probability (TP = 0.5). So, for example ABE could be followed by either ABE, CDF, ABF or CDE.

### Procedure

The experiment took place inside an electro-magnetically shielded chamber. Participants were asked to seat in a chair in front of a desk with a monitor. Their seating position was chosen so that it formed an equal side triangle with the speakers and that their eyes were at the level of the center of the screen. The experiment consisted of 6 blocks each one comprising an exposition phase of about 7 min followed by a behavioural task of about 2 min, resulting in a total duration of the experiment (including pauses) of about 1 hour. During the exposition phase, auditory stimuli were presented via the speakers while participants could watch a silent movie on the monitor in front of them. The electroencephalogram (EEG) was recorded during the whole experiment. The experimenter was present in an adjacent room throughout the experiment. He could monitor the participant’s state at all times with camera directed at the participant’s head. Participants were instructed to give a sign to the camera should they need to interrupt the experiment.

The experiment started with a set of instructions. Participants were not informed about the regularities in the arrangement of the stimuli, to ensure that any kind of learning throughout the experiment was implicit. At the same time, to ensure that participants were attentive to the stimuli, a cover task was used: The participants were asked to press the spacebar every time they heard the (higher-pitched) target sound. There were examples of the target sound in the instructions, followed by practice trials (lasting about 1 min) containing a relatively high number of target sounds. The practice trials were repeated if participants did not detect at least 80% target sounds (or had a too large number of false alarms). Participants were asked to avoid movement, especially jaws and eyes, in order to minimize artifacts in the EEG recording.

### Familiarity test and Confidence Rating

At the end of each block, a behavioral test assessed whether participants were able to distinguish two triplet sequences ending on a sound with either high or low transitional probability. Each test had 12 trials where participants were presented with three repetitions of all four possible triplet combinations (ABE vs. ABF, ABF vs. ABE, CDF vs. CDE, or CDE vs. CDF). All sequences were presented binaurally, none contained a location change, and there was a pause of 335 ms between the triplets. Participants were asked to choose which sequence sounded more familiar (pressing either “1” for the first or “2” for the second sequence). Afterwards, they rated their level of confidence about their choice of sequence (pressing from “1” – absolute unsure, could have thrown a coin – to “5” – absolutely certain). Consecutive trials did not use the same triplet root and the order of presentation of the endings was counterbalanced.

### Data Recording and Analysis

#### EEG recording

The EEG signal was recorded from 59 scalp electrodes placed in an electrode cap in compliance with the 10–10 system (see Fig. 2) at 500 Hz sampling rate using BrainAmps DC (Brain Products GmbH, Munich, Germany). Additional electrodes were placed on the left and right mastoids (the left mastoid serving as reference), on the back of the neck (serving as ground), as well as at the outer canthi of the eyes (bipolarly recording the horizontal electrooculogram [EOG]) and above and below the right eye (for the vertical EOG). All electrode impedances were kept below 5 kΩ.

#### Processing of EEG data

EEG data were analysed using EEGLAB 13^46^ within MATLAB® R2016b (The MathWorks Inc., Natick, MA). EEG data during the behavioral part of the experiment (at the end of every block) were not evaluated. EEG data were manually inspected and excluded from analysis if they contained faulty channels or periods with excessive artifacts. An Independent Component Analysis was used to remove eye and muscle artifacts. Afterwards, EEG data were re-referenced to the algebraic mean of the left and right mastoid electrodes and filtered using a 30 Hz low-pass filter (2750 points, finite impulse response, Blackman).

Samples were rejected whenever the standard deviation within a 200 or 800 ms gliding window exceeded 25 *μ*V at any electrode channel (including the EOG channels). Afterwards, data were epoched, excluding epochs following acoustical deviants or button presses (within 3 secs; i.e., rejecting activity related to the cover task of the participant). ERPs were calculated for low and high probability triplet endings with or without location change of the sound, from −100 to 400 ms relative to stimulus onset and using a 100 ms pre-stimulus baseline correction. In addition, to determine whether participants’ brain responses reflected the triplet structure of the stimulus stream, ERPs for the triplet root were calculated from −100 to 1000 ms (relative to the onset of the preceding triplet end). These ERPs were restricted to roots following high transitional probability sounds at the end of the previous triplet in order to avoid contamination of the N1 with brain responses to low transitional probability triplet ends. These ERPs did not use baseline correction but were instead 0.5 Hz high-pass filtered (550 point, finite impulse response, Blackman) in order to remove slow drifts and other low frequency trends^47^. Baseline correction was chosen for triplet endings to avoid any bias of previous activity (location change for root) whereas for roots we chose high-pass filtering as a more appropriate method in relation to root length.

**Figure 6:**
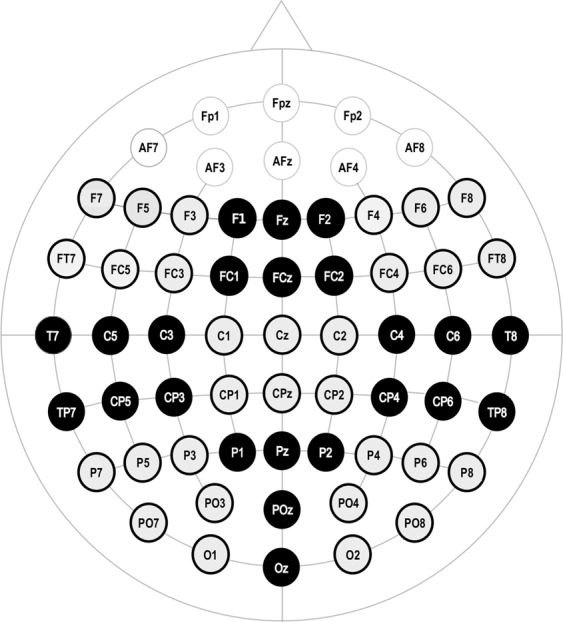
Electrodes Clusters. The electrodes were clustered in 9 ROIs which are shown in gray and black.

For statistical evaluation, electrodes were clustered into nine regions of interest (ROIs) as shown in Fig. 6, namely frontal left (F7, F5, F3, FT7, FC5, FC3), frontal middle (F1, FZ, F2, FC1, FCZ, FC2), frontal right (F8, F6, F4, FT8, FC6, FC4), central left (T7, C5, C3, TP7, CP5, CP3), central middle (C1, CZ, C2, CPZ), central right (T8, C6, C4, TP8, CP6, CP4), parietal left (P7, P5, P3, PO7, PO3, O1), parietal middle (P1, PZ, P2, POZ, OZ) and parietal right (P8, P6, P4, PO8, PO4, O2). The time windows for statistical analysis were selected in accordance with previous studies (see Introduction) and based upon visual inspection.

#### Statistical analyses

Statistical analyses were conducted using SPSS 25 (IBM Corp., Armonk, NY, USA). Behavioural data were participants’ responses to the familiarity test. Responses were classified as correct when participants chose the sequence that contained the high probability transition (and was played more frequently during exposition phase). Mean percent correct was calculated for each participant and subsequently compared against chance level (0.5; independent sample t-test, *α* = 0.05).

Three analysis of variance (ANOVAs) were conducted assessing the brain responses to the end of the triplet for either (1) statistical deviance (low and high transitional probability between triplet root and end), (2) physical deviance (standard location vs. deviant location), or (3) the combination of statistical and physical deviance. A fourth ANOVA explored the segmentation of the sound stream into the triplet structure comparing sound position within the triplet (1st, 2nd, 3rd triplet item). Each of these four ANOVAs furthermore contained two factors assessing scalp distribution: (a) scalp area (frontal, central, posterior) and (b) lateralisation (left, midline, right) as well as one factor assessing development over the course of the experiment: (c) experiment block (1 to 3; the first, middle and last two blocks of the 6 blocks of the experiment were grouped in order to obtain a better signal-to-noise-ratio).

## Supplementary information

Supplementary Information

## Data Availability

The datasets generated during and/or analysed during the current study are available from the corresponding author (Barbara.Tsogli@uib.no) on reasonable request.
