# Author Correction: When the statistical MMN meets the physical MMN

**DOI:** 10.1038/s41598-019-52009-8

**Published:** 2019-11-05

**Authors:** Vera Tsogli, Sebastian Jentschke, Tatsuya Daikoku, Stefan Koelsch

**Affiliations:** 1 University of Bergen, Department for Biological and Medical Psychology, Postboks 7807, 5020 Bergen, Norway; 2 University of Bergen, Department of Psychosocial Science, Postboks 7807, 5020 Bergen, Norway; 3 Max Planck Institute for Human Cognitive and Brain Sciences, Stephanstr. 1a, 04103 Leipzig, Germany

Correction to: *Scientific Reports* 10.1038/s41598-019-42066-4, published online 03 April 2019

This Article contains errors.

In the Results section under the subheading ‘ERPs of Triplet Endings’,

“Pairwise comparisons examined how the effect of transition probability evolved across the experiment (see Fig. 2C).”

should read:

“Pairwise comparisons examined how the effect of transition probability evolved across the experiment.”

In the Results section under the subheading ‘Physical Deviants’,

“Triplet endings with location changes elicited consistently larger negativities throughout the course of the experiment even though the amplitude size diminished over blocks (see Fig. 3C).”

should read:

“Triplet endings with location changes elicited consistently larger negativities throughout the course of the experiment even though the amplitude size diminished over blocks.”

Additionally, the key in Figure 2 is incorrect.

“Low Prob. with Physical Deviance”

should read:

“Low Probability”

and

“High Prob. without Physical Deviance”

should read:

“High Probability”

The correct Figure 2 appears below as Figure 1.

**Figure 1:**
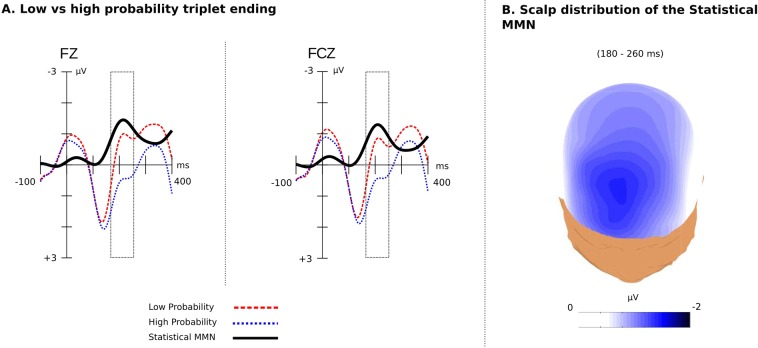
.

