# Supplementary material for: When the statistical MMN meets the physical MMN
Source: Sci Rep. 2019 Apr 3;9:5563. doi: 10.1038/s41598-019-42066-4 (PMC6447621; doi:10.1038/s41598-019-42066-4)
Supplement: Supplementary file 1 — Supplementary Information [file 41598_2019_42066_MOESM1_ESM.pdf]

Supplementary Information

Article:

When the statistical MMN meets the physical MMN

Vera Tsogli, Sebastian Jentschke, Tatsuya Daikoku, and Stefan Koelsch

Word segmentation

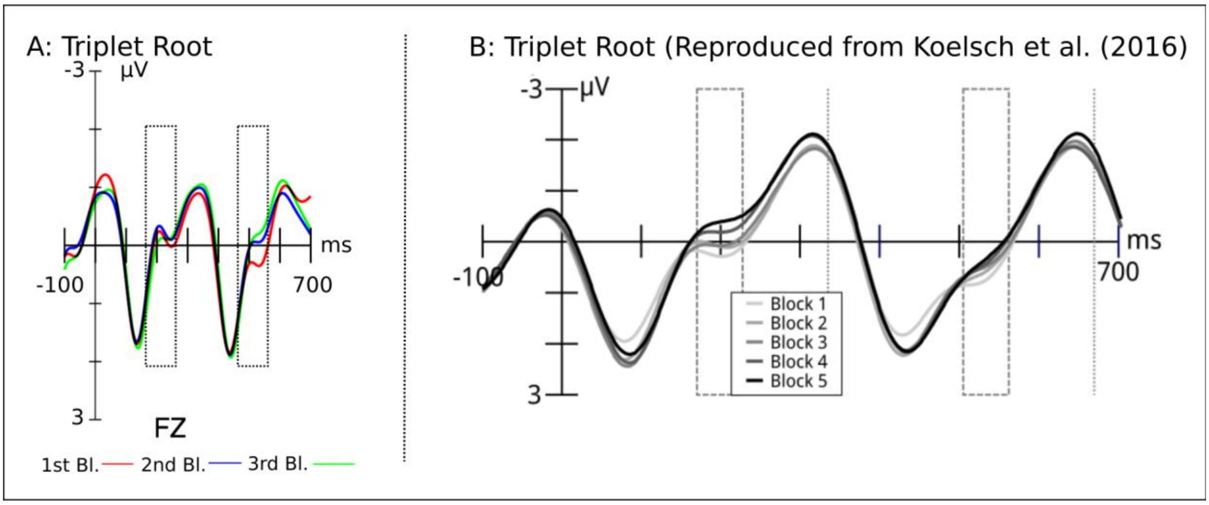

Figure 1. Mean ERP results for triple roots Mean ERP waves for triplet roots (i.e., first and second items of the triplet), recorded at Fz (-100 – 700 ms relative to the onset of the triplet root), separately for each block. The dashed rectangles indicate the time window used for the statistical analysis. **(A)** Mean ERP traces from the current study where no significant word segmentation effect was observed **(B)** Mean ERP traces from the study by Koelsch et al. (2016) where significant word segmentation effect was observed.

Statistical MMN topographies

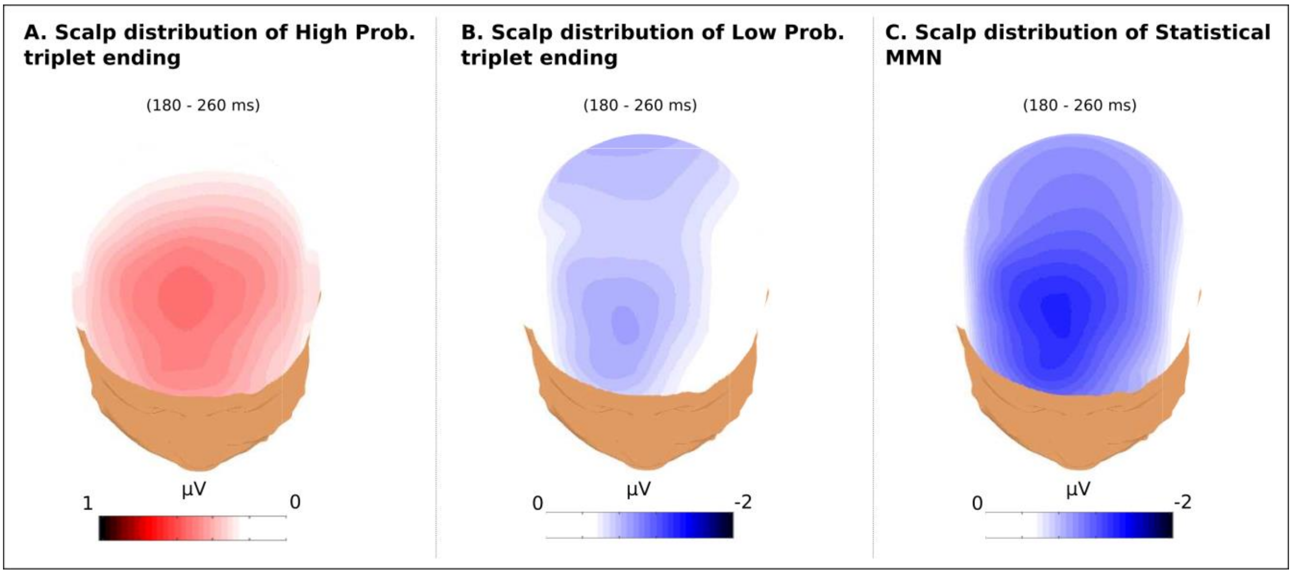

Figure 2. Scalp topographies for the Statistical MMN. (A) The scalp distribution of the high probability triplet ending over the time window (180 to 260 ms). **(B)** The scalp distribution of the low probability triplet ending over the time window (180 to 260 ms). **(C)** The scalp distribution of the statistical MMN over the time window (180 to 260 ms) for triplet ending.

Physical MMN topographies on the low probability ending triplet

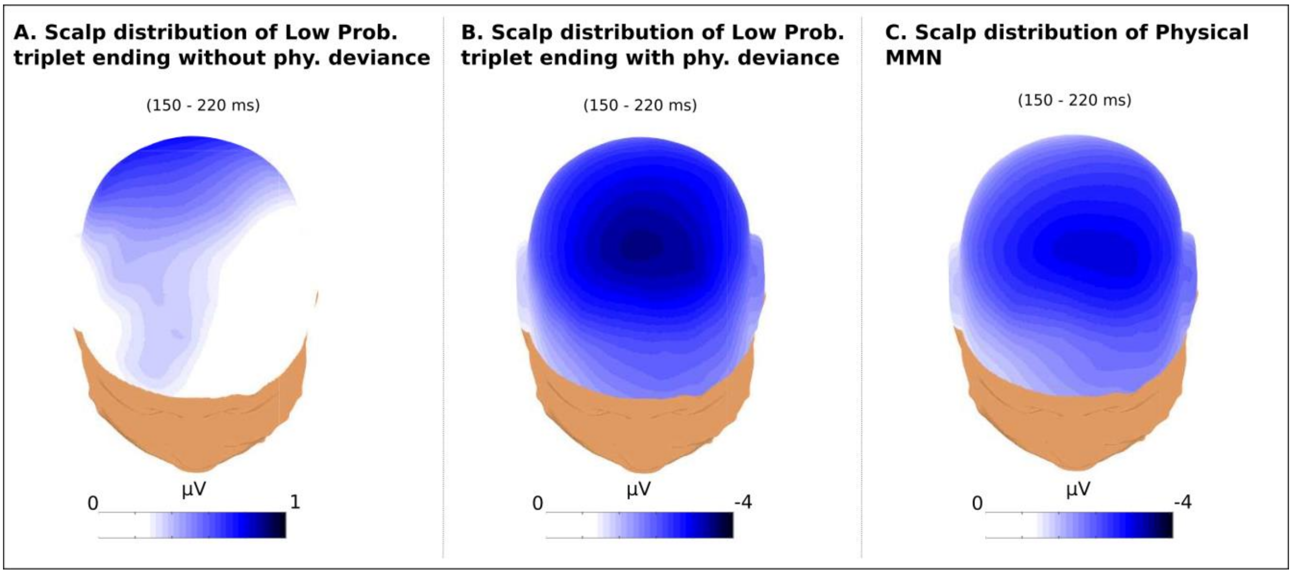

Figure 3. Scalp topographies for the physical MMN on the low probability ending triplet. (A) The scalp distribution of the low probability triplet ending without physical deviance over the time window (150 to 220 ms). **(B)** The scalp distribution of the low probability triplet ending with physical deviance over the time window (150 to 220 ms). **(C)** The scalp distribution of the physical MMN over the time window (150 to 220 ms) for triplet ending.

Physical deviance effect on the high probability triplet ending

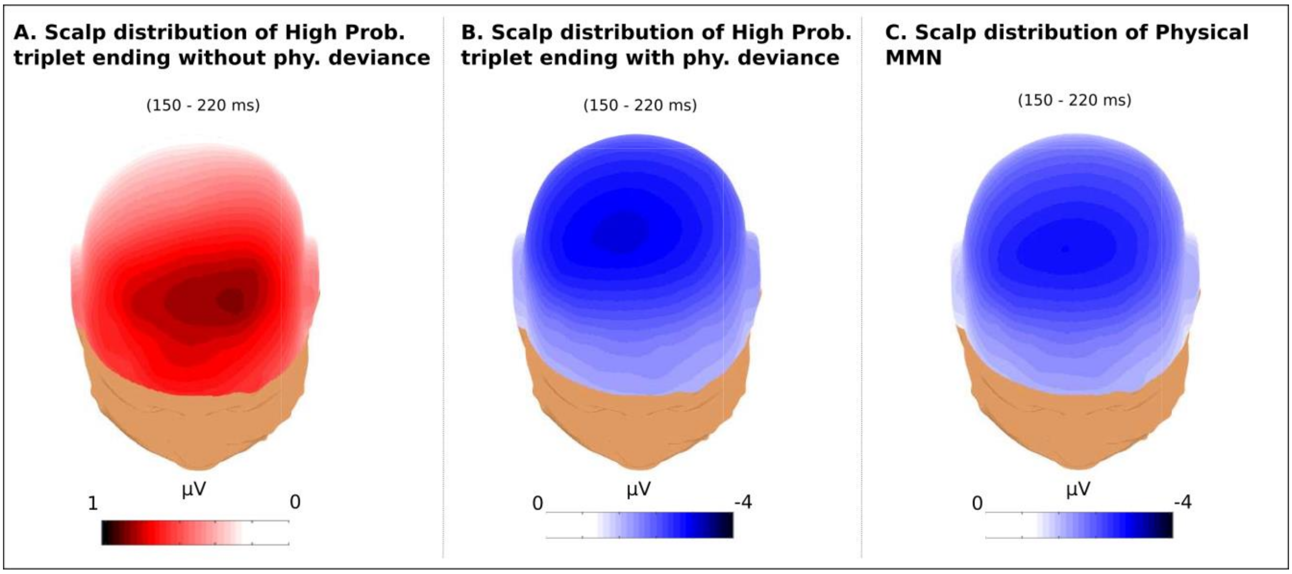

Figure 4. Scalp topographies for the physical MMN on the high probability ending triplet. (A) The scalp distribution of the high probability triplet ending without physical deviance over the time window (150 to 220 ms). **(B)** The scalp distribution of the high probability triplet ending with physical deviance over the time window (150 to 220 ms). **(C)** The scalp distribution of the physical MMN over the time window (150 to 220 ms) for triplet ending.

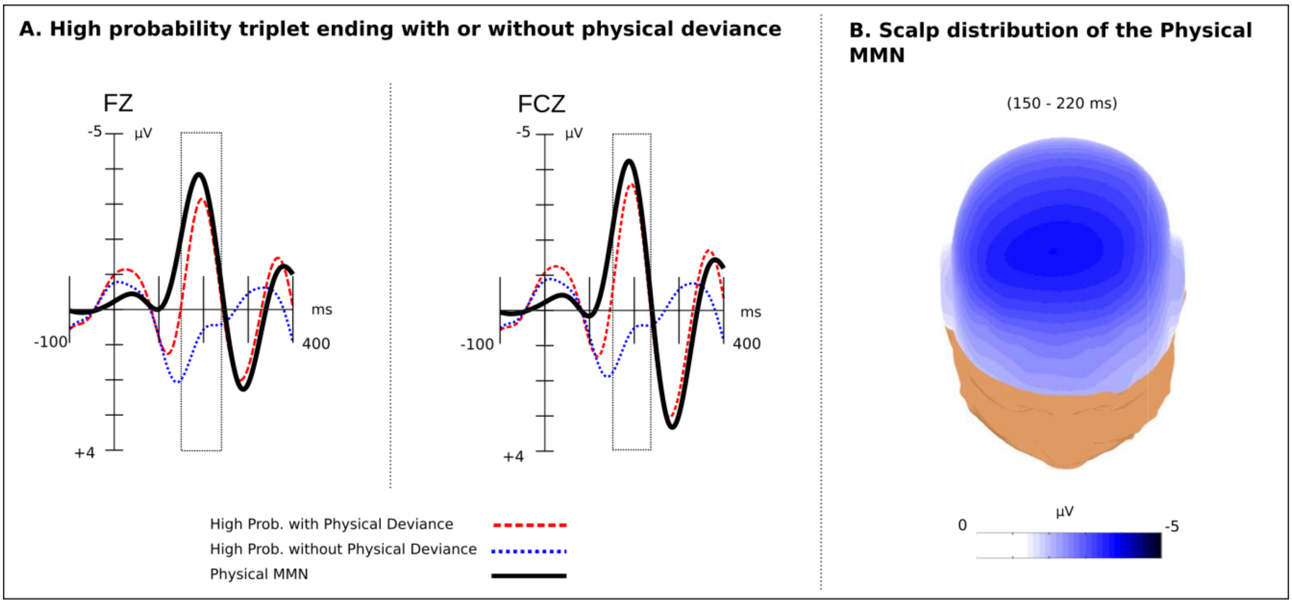

Figure 5. The physical MMN (phMMN) on the high probability triplet ending. (A) The phMMN (black line) generated due to the violation of sound location of triplet ending from the "standard" to the "deviant" side, as captured at electrodes Fz and FCz. Last tone (triplet ending) onset is at 0 ms and has a duration of 300 ms. ERPs are baseline corrected 100 ms prior to the onset of the trigger. **(B)** The scalp distribution of the phMMN over the time window (150 to 220 ms) was maximal over the frontal and central midline regions.

P3b Scalp Topographies

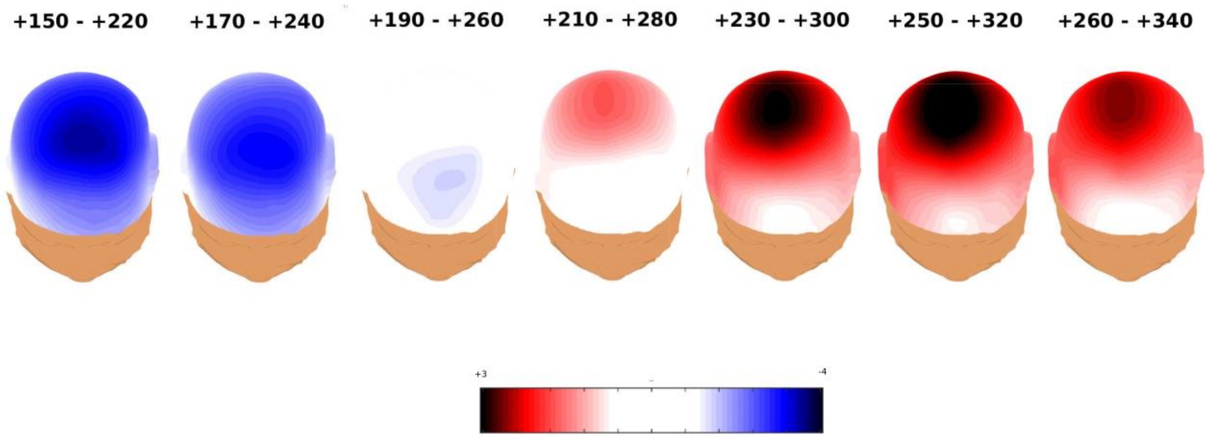

Figure 6. The P3b effect. Scalp topographies for the distinct P3b which followed the physical MMN and reached a peak over the time window from 150 to 320 ms.
